# Agency, Goal-Directed Behavior, and Part-Whole Relationships in Biological Systems

**DOI:** 10.1007/s13752-023-00447-z

**Published:** 2023-11-08

**Authors:** Richard Watson

**Affiliations:** Institute for Life Sciences/Electronics and Computer Science, University of Southampton, Southampton, UK

**Keywords:** Agency, Complex systems, Learning, Levels of organization

## Abstract

In this essay we aim to present some considerations regarding a minimal but concrete notion of agency and goal-directed behavior that are useful for characterizing biological systems at different scales. These considerations are a particular perspective, bringing together concepts from dynamical systems, combinatorial problem-solving, and connectionist learning with an emphasis on the relationship between parts and wholes. This perspective affords some ways to think about agents that are concrete and quantifiable, and relevant to some important biological issues. Instead of advocating for a strict definition of minimally agential characteristics, we focus on how (even for a modest notion of agency) the agency of a system can be more than the sum of the agency of its parts. We quantify this in terms of the problem-solving competency of a system with respect to resolution of the frustrations between its parts. This requires goal-directed behavior in the sense of delayed gratification, i.e., taking dynamical trajectories that forego short-term gains (or sustain short-term stress or frustration) in favor of long-term gains. In order for this competency to belong to the system (rather than to its parts or given by its construction or design), it can involve distributed systemic knowledge that is acquired through experience, i.e., changes in the organization of the relationships among its parts (without presupposing a system-level reward function for such changes). This conception of agency helps us think about the ways in which cells, organisms, and perhaps other biological scales, can be agential (i.e., more agential than their parts) in a quantifiable sense, without denying that the behavior of the whole depends on the behaviors of the parts in their current organization.

## Introduction

Informal accounts of organisms and their behavior often include agential language, such as choosing an action in order to, or for the purpose of, attaining some goal or aim, or with an intention to achieve some desired state. In contrast, formal accounts of organisms and their behavior often eliminate such language in favor of reductionist, mechanistic accounts (Sultan et al. 2022). Whilst higher-level descriptions of complex systems are sometimes accepted as pragmatically useful in some circumstances, including taking an “intentional stance” (Dennett 1987) where appropriate, these are often assumed to be inferior to reductionist mechanistic accounts. Some take this to mean that accounts based on agential notions are explanatory shortcuts, and there must always be, in principle, a way to explain (or explain away) agential behavior in mechanistic terms. In organismic and evolutionary biology, extreme forms of the latter may refer exclusively to genes (and their downstream consequences) as explanations for traits or behaviors (Sultan et al. 2022).

Somewhat curiously, agential language is sometimes invoked for genes too (e.g., “selfish genes”; Gardner and Welch 2011), or more exactly, for the evolutionary process acting at the genetic level, as if the evolving gene chooses among options in order to satisfy its aim of, or to solve the problem of, maximizing its survival and reproduction. Although this is generally excused as mere metaphor, note that the move from game theory (to explain the behavior of deliberative strategic agents) to evolutionary game theory (to explain changes in allele frequencies within a population) explicitly equates agential and evolutionary framings (Maynard Smith 1986). Perhaps this is because agential framings satisfy the explanatory aim of identifying a “prime mover” in biological systems. The real issue might be not so much an argument about the validity of agential framings per se but an argument about which level of biological organization is the legitimate owner of such agency.

Some have argued that, for some important aspects of biology, agential concepts at the organismic level provide explanations that cannot be provided by gene-centered accounts (Sultan et al. 2022). For example, phenomena such as inclusive inheritance, phenotypic plasticity, and the origin of novelty are poorly explained by accounts that refer only to differential selection on allelic variants, and are better explained by acknowledging the active role of organismic phenotypes in the evolutionary process (Moczek et al. 2011; Sultan et al. 2022). A notable common factor is that each of these cases involves either (1) modifications to one or more of the core mechanisms of the Darwinian machine (inheritance, variation and selection), (2) the supposed independent autonomy of these mechanisms, or (3) introduces some fluidity in the identity of the unit to which these mechanisms apply (Uller and Helanterä 2019; Watson and Thies 2019). These phenomena thereby make it difficult to construct, even in principle, an evolutionary explanation based solely on the gene and the maximization of its survival and reproduction. Should we just accept that the mechanistic details are complicated, or is a different kind of causal story needed? Can these issues be treated as rare exceptions, or do they indicate that a perspective eliminating agency, or limiting prime movers to only at the lowest organization scale (e.g., genes), is fundamentally lacking?

It is easy to understand why explanations that start with lower-level parts, i.e., bottom-up or “component-to-system” explanations, might be favored (Sultan et al. 2022). Understanding seems to necessitate the building-up of an explanation from more basic concepts that are already understood, so for a complex system, it seems natural to break it into parts and explain their working. In this view, a system is assumed to be nothing more than what its parts do (in interaction with one another). In contrast, agential explanations seem to start with an intention, goal, or target function for the system as a whole and from this explain why the parts have the form they do, i.e., top-down or “system-to-component.” But the presupposition of such a goal or function seems to come from nowhere—an explanatory “skyhook” (Dennett 1996).

In what follows, we aim to present a way of characterizing agency at a particular scale of organization. We will suppose (without prejudice) that a system admits a mechanistic account in principle. That is, if all the relevant microscale state variables are known then the inner workings of an agent, like any other system, afford a reductionist, mechanistic account (even if this might not be very useful in practice). Whilst we are not committed to the existence of such a microscale mechanistic account in all cases, we aim to say something useful about the agency of a system even when such an account is available (for example, when an empirical experiment verifies the mechanism underlying a behavior). Consistent with this, we avoid trying to identify any vitalist criterion that categorically distinguishes between agential and non-agential (“ordinary mechanistic”) systems in absolute terms. Instead, we offer a definition of agency in quantifiable but *relational* terms—more specifically, a *scale-relative* notion of agency. In essence, our approach is to focus on whether the agency of the whole is greater than that of its parts. For example, is the agency of the multicellular organism greater than that of the cells it contains? This is a legitimate question regardless of whether one considers the parts or the whole to be obviously agential or obviously non-agential with respect to some absolute criterion (it can also be considered a separate issue from whether or not a particular stance is a pragmatic explanatory heuristic). However, it will mean that the agency exhibited by a system will be sensitive to different ways of defining its subagents.

Our approach is to discuss the internal organization of a system (the functional relationships among its parts), and how changes in this organization affect the behavior of the system as a whole and the resolution of frustrations among its components. We build up to this by addressing the following questions:

- In what way can a system have behaviors that belong properly to the system (not to the smaller parts of which it is composed nor to the conditioning of its larger context)?

This involves discussion of how a system can have behaviors that belong to the whole system (not decomposable into the behaviors of its parts), and in what way the organization necessary for such non-decomposable systemic behaviors can also have a non-decomposable systemic origin. The latter requires that the system’s organization is not fully determined by its initial internal conditions (construction or design), and also under-determined by its subsequent external conditioning (training or selection) in the sense that the result of any training or selection also cannot be decomposed into the training or selection of its parts.

We then address the more specific possibility that these systemic behaviors have the effect that the agency of the system is greater than the agency of its parts:

- What does goal-directed behavior mean from a dynamical systems perspective? And how does the goal of the whole relate to the goals of its parts?
- And relatedly, when comparing the behavior of a system with one organization to that of the same parts in a modified organization, in what sense can the modified system do something *better* than the original system rather than merely doing something *different*?

This builds into a notion of agency based on the idea of a system that exhibits goal-directed behavior that is greater than that of its parts. That is, a system has increased in agency if its current organization enables its parts to do what they want to do better than they could with the organization that it had previously. At the system level this will appear as a system that exhibits goal-directed behavior in the sense of delayed gratification—taking trajectories that forego short-term gains (or sustain short-term stress or frustration) in favor of long-term gains. This requires that the organization of relationships among its parts constitutes distributed systemic knowledge of the system’s potential behavior and resulting frustrations in the given environment, such that the modified relationships contain information that changes outcomes for the better. This cognitive or “intelligent agent” sense of agency (Okasha 2023, 10.1007/s13752-023-00439-z.) may seem too special for many types of biological systems of interest. But we support the position that problem-solving, construed in an appropriate space of possibilities, can be identified as a fundamental feature of all organisms (Fields and Levin 2022). In particular, we make the case that the notion of an agent that is “more than the sum of its parts” implies some problem-solving competency with respect to the frustrations that its parts experience (and that this competency is under-determined by the conditioning of its larger context).

Reconnecting with the first two questions, we discuss how a system might acquire the necessary organization to exhibit agency through experience, i.e., learning, without presupposing that this is an aim for the system or, indeed, that the system is (already) an agent that can have an aim. This perspective emphasizes the importance of generalization and inductive learning as necessary mechanisms for goal-directed behavior, and also (thereby) to support the idea that the salient properties of an agent can be a product of its own systemic history and not of its parts nor its contextual conditioning (i.e., produced by *the history of the system, the whole system, and nothing but the system*).

## Towards a Scale-Relative Notion of Agency

In what way can a system have behaviors that belong properly to the system (not to the smaller parts of which it is composed nor to the conditioning of its larger context)?

The way a system works (now) is a different matter from how a system came to be. *How does it work?* is a different question from *Why does it work that way and not some other way*? Even when the “how” question can be satisfactorily explained with a component-to-system account (the mechanistic assumption), the “why” may require a system-to-component account.

Consider the parts of an organism, such as the organs of a multicellular organism. For the moment, let us help ourselves to the assumption that the function of the organism as a whole is the survival and reproduction of the organism. If we also take for granted the organization of the parts as we find them now, and thus the way in which they decompose this overall function into subfunctions, then it is possible to account for the functioning of each part in its context, and hence the functioning of the whole. Explaining how the organism works *as we find it* can thus begin with the smaller parts of which it is composed, a component-to-system account. For the sake of the argument, let us grant that we can do this at the level of organs, tissues, cells, or molecules as we wish. However, when we want to explain why the organization of the organism is like this and not some other way, simply saying that this was necessary to facilitate the survival and reproduction of the whole, i.e., determined by the conditioning of its larger external context, is inadequate. There are, after all, many ways to survive and reproduce, not all of which have the same parts in the same organization.

Here we generally retreat to a historical explanation, for example, because this way of doing it conferred an incremental advantage in organismic function from what came before. Given that not all branches of the tree of life resulted in the same convergent outcome, divergences would be attributed to differences in external conditions. With this historical context in mind, it is tempting to decompose the historical explanation of the whole into a historical explanation of each part. Can we simply assume that the organization of the whole is explained by the historical account of each part? For example, we might argue that this part has the particular form it does because this form conferred an advantage in organismic function from what came before *all other things being equal*. However, this ceteris paribus approach implicitly assumes the organizational context the part finds itself in now. But this organization, without which the meaning of the part is not well-defined, has (still) not been explained.

For example, when we think about explaining organisms by decomposing them into smaller parts, such as genes, we invariably see reference to system-level functions, such as fitness advantages, and sometimes also the context of other parts, for instance, because this allele confers a fitness advantage *in this genetic context*. Naturally, we understand that a dependency on genetic context is always present; but how much context is needed to disambiguate the function of the part? Is this part sensitive to *one* other part or *all* the other parts? When we unpack a biological explanation, this naturally introduces more context. For example, “this gene came to have the form it does, and not some other, because that form increases light sensitivity in the retina, because that enables the organism to see better, because that enables it to avoid predators better, because that enables it to survive better, (all the way to the ultimate cause) because that enables it to leave more offspring.” Of course, in some other context, such as when that gene is in the context of an organism that lives underground, the circumstance may confer the opposite result for selection on the same allele. Moreover, the environment in which an organism lives is not an entirely exogenous parameter but a variable that is itself determined by the behavior of the organism (Laland et al. 2015). Accordingly, without the context created by the organism itself we are left with essentially zero explanatory power to answer why this part has the form it does or, therefore, how it came to take that form. This leaves us unable to explain the organismic organization by decomposing the evolutionary history of the system into the evolutionary history of each part.

**Fig. 1:**
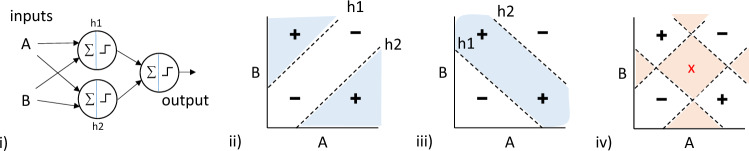
An artificial neural network illustrates the under-determination of component organization and of system function. **i** A network consists of two inputs (A and B), two hidden nodes (h1 and h2), and one output node. Each node computes a nonlinear (thresholded) weighted sum of its inputs (arrows). **ii** The feature space describes the mapping from the inputs to the output (shaded = output high, unshaded = output low). Each hidden unit corresponds to one linear decision boundary in this space (dotted lines). The four points indicated by “ + ” and “−” are training examples; inputs that are in the target class and not in the class, respectively. Here the target function is A-XOR-B, logical Exclusive-OR. This function is *nonlinearly separable* because there is no possible single linear boundary that separates negative from positive training samples. For the h1 and h2 boundaries shown in this example, an output computing h1-AND-h2 correctly classifies all four training samples. **iii** This is a different example where an output computing h1-OR-h2, over different h1/h2 decision boundaries, also correctly classifies all four training samples. Together these two examples show that any hidden unit can have multiple functional roles, i.e., it is under-determined by the target function (Box 1). **iv** Although (**ii**) and (**iii**) both make no errors on the training set, they compute different functions (blue shaded regions). In fact, these two different networks only agree on half of the feature space and disagree on the other half (shaded red). This means that the two networks generalize differently and can give the opposite classification for a new input, such as point “x”

The functional role of each part becomes *entirely* context dependent when the function computed by the parts is *non-decomposable*; here the functional role of (and hence the direction of selection on) one part depends on the value of another and vice versa. Box 1 discusses a worked example of a minimal system (two components and one interaction), specifically, an artificial neural network with two interneurons combined by one output neuron (Fig. 1). We see that each part can have *any* functional role; it is entirely under-determined by the function computed because the parts mutually define their functional roles in complement to one another. We can also use this simple example to illustrate that the organization of the system induced by training *under-determines* its systemic behavior (as well as its internal organization). That is, although the multiple organizations are equivalent insomuch as they each confer zero error on a training set, they do not compute the same function and are therefore not equivalent with respect to their *generalization* properties. It is exactly this generalization that matters for learning systems, not their ability to memorize the training set. Moreover, the generalization they exhibit is only very weakly dependent on initial conditions and strongly dependent on systemic symmetry-breaking dynamics. This means that, with respect to the generalization that matters, the training of the system cannot be decomposed into the training of the parts. Thus, the behavior of the system depends on the history of the system, the whole system, and nothing but the system (Box 2).

The under-determination of a system’s organization is all the more problematic when we consider that the overall function of an organism—its very status as an evolutionary unit—is also dependent on the organization of its parts as we find them now. The cells of which the multicellular organism is composed have an evolutionary history that is predominantly unicellular. In each of the evolutionary transitions in individuality, “entities that reproduced independently before the transition reproduce only as part of a larger whole after the transition” (Szathmáry and Maynard Smith 1995; Watson et al. 2022). So, it is not just that a component-to-system account fails to explain why the survival and reproduction of the organism is achieved one way and not some other way, it cannot even explain why the organism is an evolutionary unit in the first place and hence a legitimate entity to possess such a function. The assumption that the function of the organism as a whole is the survival and reproduction of the organism is thus helping itself to too much, not least the evolutionary status of the organism as an entity that can have a fitness.

Thus, a bottom-up (component-to-system) account might explain *how* something works. For the “how” question, it may be sufficient to take the organization of the system as a given, and then explain how the parts contribute to the whole *given that organization* (even if it has non-decomposable functions). In contrast, explaining *why* its parts have the form that they do can require a system-to-component account involving the whole system. It might not be possible to explain its organization by decomposing the evolutionary history of the system into the evolutionary history of each part because the direction of selection on each part depends on the holistic history of the system (Box 2). In this sense the system’s behavior, and the organization that produces that behavior, can belong properly to the system insomuch as it depends on the holistic history of the system—not its smaller component parts nor its larger external context.

This non-decomposability is thus crucial to identifying properties that belong to a system over and above its parts, and that are not given by its construction or design, nor by its subsequent training or selection, but by its holistic history. Having established that a system can have its own behaviors in this formal sense, next we apply these concepts to assess whether these behaviors are agential. That is, we ask whether a system can have goal-directed behavior over and above the goal-directed behavior of its parts.

What does goal-directed behavior mean from a dynamical systems perspective? And how does the goal of the whole relate to the goals of its parts?

A hallmark of intelligent behavior is the idea of overcoming or going around obstacles. If it is not possible to go directly to a particular destination (e.g., as per a least action principle), it may be possible to take a different route that goes further away from the goal in the short term, in order to get closer to it in the long term. In psychology, the equivalent concept is delayed gratification (e.g., the “one marshmallow now or two later” test).

We discuss this notion in the context of a dynamical system minimizing an energy function. This begins by describing a dynamical system that behaves in an entirely ordinary (conventionally non-agential) way and then builds up to one that behaves in a goal-directed way. In the style of Fields and Levin (2022), we consider a configuration space (a set of states and a neighborhood), and a landscape over that space, such that behavior is a local minimization (gradient descent) process over that landscape. More specifically, we define this landscape as a set of interactions or constraints over a set of parts. Because we want to ensure we arrive at a concept of agency that is fully compatible with a mechanistic account, we skate very close to a system that is—by any reasonable definition—not agential at all. But we think this is the right end of the spectrum to start from.

Consider a ball on a slope acted on by gravity. In responding to the force of gravity, and the reaction from the local gradient, the ball’s lateral movement is in the direction that lowers its vertical height—it goes downhill. The end point of this process depends on the start point and the shape of the landscape only. If a ball arrives at a location where the local gradients in all directions are upwards (a local minimum) then that is the end of the trajectory. No need to invoke agential notions for this type of behavior, of course. Note that there may be points that are lower in height elsewhere on the landscape, but the local gradients do not permit movement toward these. That is, the ball is “stuck” or obstructed from going further downhill.

This local minimum is a result of constraints. For example, for a two-dimensional landscape over variables x and y, the ball naturally moves laterally in the downhill direction of x, and in the downhill direction of y. If movements in all the dimensions of the landscape were independent, it would always be able to go downhill until all dimensions were minimized. But if movements in one dimension are not independent of movements in another then going downhill in one dimension might mean that it goes uphill in another. If the net effect is upward, this interdependence prevents further movement in either dimension. It is the interactions or constraints between the x and y variables (different dimensions of movement) that cause local optima (a.k.a. sign epistasis in a fitness landscape; Weinreich et al. 2005), or constraints in a constraint optimization problem.^1^ Note that it is already possible to determine that one point in configuration space is better than another with respect to satisfying these constraints. That is, some local optima resolve more constraints than others. For a system to do better than merely finding the local optimum, i.e., moving to a point that resolves more constraints than the local optimum, requires the system to go uphill in a specific direction for a while (“patience”; Fields and Levin 2022), i.e., to “go the long way around.”^2^

If you see an object that, unlike the ball, occasionally moves *up* local gradients, what do we make of this? Clearly the external forces acting on the ball (gravity and the local slope) cannot be the whole story. One way or another there must be other forces acting on the system that we were not taking into account. This need not be complicated. For example, if we include momentum, then the state of the world is not represented fully by gravity and the slope given by *position*; we also need the *velocity* (and mass, which we will assume is constant). When all these variables are taken into account they fully describe the trajectory of the ball—including the periods where potential energy is converted back and forth to kinetic energy, occasionally causing it to go uphill.

Suppose instead some of the variables remain obscured or “screened-off” from observation. Let us take the external variables (position, slope, gravity) to be observables and the other variables (momentum) to be “internal” or unobservable.^3^ We note that the uphill behavior of the ball is not explained by the external observables but *is* explained when the internals are also taken into account. This screening-off is just a point of view (or “stance”) that seemingly causes surprising behavior, but only because some of the necessary variables were not described/observable. We thus adopt a concept of agency that is observer dependent (Fields and Levin 2022), or more exactly, dependent on what is observed, i.e., not just that agency is subjective or “in the eye of the beholder,” but that it depends on what variables are observed and which are not. This retains compatibility with a materialist assumption. It also aligns with the most basic of agency concepts, i.e., of a system caused to act by “its own” internal state rather than external factors (Okasha 2023, this volume). But we can develop this into something much more specific and useful.

With this cut between internal (unobservable) and external (observable) variables we can ask, what kind of internal state causes an external behavior that is agent like? Clearly, the behavior of the system taking into account its internal state can be different from that expected if we did not take internal variables into account. But for agency, it is not sufficient simply that the behavior is *different*—it needs to be *better*. To develop this we return to the notion that the ball went downhill but got stuck at a local optimum by constraints that prevented it from going *further* downhill. Specifically, we assess whether the internal state confers on the system an ability to go downhill further than it does without that internal state—to “go around obstacles” insomuch as it finds configurations that better resolve constraints, or “delays gratification” insomuch as by this action the constraints are better resolved in the long term than they would be via the “obvious route,” i.e., without such modification. To the extent that this involves modifications to trajectories that are specific to the particular features of the landscape (not, for example, provided by stochastic perturbation), we will say that the internal variables constitute information about the external world, and we can quantify the utility of this information and its deployment by measuring its effect on the ability of the system to go downhill further than it does without that internal state. That is, if the system has an internal state that causes all of the parts of the system to do what they were already doing *but better*, then we will say that the system has knowledge of the external world, and in deploying this information to modify its behavior, the system is not at zero on the agency scale.^4^

An objective function over a state space (a landscape) defines the superiority of one point over another, and makes the idea of going down further than a local optimum well-defined. Logically, in order for a physical system to go somewhere different from the nearest locally optimal solution in configuration space, the energy function (describing the dynamics of the system) must be different from the objective function (describing the quality of solutions to the problem). The former is a behavior that includes the unobservable or “subjective” variables internal to the agent, and the “objective” function does not. Then going to the local minimum in *Function2* (with internal variables), takes us to a configuration that is better than the local optimum in *Function1* (without this agential influence). In principle, *Function2* could be arbitrarily (un)related to *Function1*—causing the system to exhibit any behavior imaginable. But saying that the system goes further downhill is specific—it requires a system that avoids local minima (obstacles, or short-term gains) in the objective function in order to find optima that are lower in the objective function (better long-term gains). In this sense, it is not just different but superior.

Nonetheless, this description depends on the assertion that *Function1* was an objective function—*the real problem*—and *Function2* was a way to solve *Function1*. Is this observer dependent (Fields and Levin 2022)? It could be argued that the expected behavior without internal variables is not an objective function nor a problem to be solved—it is just the energy function of a different system, namely the system without the internal variables. In this case, using *Function2* to move to configurations that are lower in energy than local optima (in *Function1*) is not *better* in any objective sense than moving to local optima (in *Function1*)—just *different*. In the following subsection, however, we will address this issue and show how the distinction between an energy function and an objective function corresponds to the distinction between the behavior of the whole rather than its parts.

At one extreme, minimal agency in this framework still skates very close to no agency at all. For example, a ball with momentum actually can find better optima compared to a ball without momentum (in some environments). If the slope that the ball has been travelling on is, more often than not, moving toward points that are lower than the local minimum, then the momentum may enable the ball to get out of the local minimum (go over a small obstacle) in the direction of a better one. The truth of the “more often than not” caveat depends on the properties of the landscape. By “experiencing” the landscape, the ball accumulates over time some information about the landscape and “deploys” that information at the next moment in time. The more consistent the slope over the past experience, the more momentum it has to overcome a local obstacle should it encounter one, and if that past experience is representative of slopes to come, then that will enable it to find lower points in the long term. But even if this is positive information gain, momentum cannot know very much about the world because it is a single directional quantity—"go *this* way.” It cannot accumulate detailed information over longer timescales because the most recent momentum is the only momentum it can know—if the ball changes direction, all history beyond this backwards time horizon is lost.

At the other extreme, we could imagine an intelligent agent with detailed internal knowledge of the world. With this knowledge it can act against the indications of the immediate external environment but in a particular way that enables it to do “better.” In particular, it does not merely go down to the nearest local minimum (nor does it go stochastically uphill in random directions). Rather it goes uphill in a specific direction that leads to lower points in the long term. It *knows which way to go*. This is, we think, what it means to say that a system’s behavior is *goal-directed* and ties directly to an “intelligent agent” sense of agency (Okasha 2023).

Note that this is a matter of degree—a little bit of knowledge about the world can be put to work to deviate a little bit from what is “naturally downhill” (i.e., according to the external forces acting on the system). A system with a lot of knowledge can go on long, temporally extended excursions, transiently visiting specific points that look terrible in the short term but lead to somewhere better in the long term.

When comparing the behavior of a system with one organization to that of the same parts in a modified organization, in what sense can the modified system do something better than the original system rather than merely doing something different?

When we speak of moving “towards a goal” or “improvement” or “better” we need to define what metric of quality we are using. What frame of reference do we have to avoid this being arbitrary? If *System2* (e.g., with internal variables X, and hence energy *Function2*) does something different from *System1* (with no internal state, and energy *Function1*), who is to say whether that was “better”? Perhaps it is just different. In biology, this need for a normative frame of reference is usually resolved by appeal to an absolute metric of quality or behavior; specifically, behaviors that (one way or another) support the survival and reproduction of the organism. This ties any notion of agency to evolution by natural selection and Darwinian fitness. In an attempt to be more general (such that one might, for example, assess the agency of hurricanes or societies or ecosystems), agency is sometimes tied to self-preservation, persistence, or such like (the differential survival aspect without the differential reproduction aspect). Whilst this attachment to ultimate goals of survival and reproduction is understandable in biology, it seems unnecessarily limiting.

Here we suggest an alternative approach that concerns the fundamental individuality of the agent. Instead of describing whether the behavior of the whole is good for the whole, we examine whether the behavior of the whole is good for the parts. More exactly, we examine whether the behavior of the whole when organized (i.e., with a specifically modified internal state) is better for the parts than the behavior of the whole when disorganized (i.e., with an arbitrary or original internal state). This seems like robbing Peter to pay Paul—discharging the need to define a normative behavior for the whole only by incurring the need to define a normative behavior for the parts. But this move will be useful—especially for thinking about biological organisms and whether they are “more than the sum of their parts.”

To unpack this idea, imagine that the system is not one ball (on a multidimensional landscape) but many different balls on many different (one-dimensional) slopes. If each of the slopes is simple and all the balls/slopes are independent, then the whole system can reach the global minimum—all the balls at the bottom of their respective slopes simultaneously. But if there are interactions or constraints between the behavior of one and the behavior of another, this is when the system can get stuck—some of them are “happy” and some of them are not (a.k.a. *frustration* in the context of physical complex systems, or *stress* in biological complex systems). That is, some of them are in configurations where the interaction between them is resolved and others are unresolved, or in tension. The quality of a configuration is defined in terms of how many constraints are resolved (or total system energy, or total system stress). This is still not quite an objective norm, however—because who says that balls are *supposed* to go downhill further? Maybe they are *supposed* to get stuck. That is what they do, after all. Who says stress is abnormal, and not just some other state a cell or an organism can be in?

Nonetheless, even though it requires us to presume a normative behavior for the parts, we suggest that quantifying how the behavior of the whole objectively relates to the normative behavior of the parts is a meaningful approach. Specifically, it enables us to quantify whether the agency of the whole is greater than the agency of its parts. Whatever the goals of the parts, a configuration for the system as a whole can be better or worse with respect to satisfying those goals. Even if the goals of the parts were defined arbitrarily, goal-directed behavior can be quantified objectively at the system level *given the goals of the parts*.

This can be quite natural. For example, if the goal of each ball is to go downhill then one system configuration is objectively better than another if more of them get to lower configurations (or a reduction in total height). This is then a specific case of the more general scenario where the goal of each component is to resolve the constraints acting on it from others and the environment, and a system configuration is better (in that environment) if more of those constraints are simultaneously satisfied. With this perspective, we substitute a notion of normative behavior for the system as a whole with a normative behavior for each of the parts and the set of constraints between them.

This approach trades the question of agency in absolute terms for the question: *how does agency scale from one level of organization to another*. We think this is a good compromise if we take agency to be intimately related to individuality (e.g., in the sense of an organism being “more than the sum of its parts; Watson et al. 2022). It does not offer an essential or vitalist boundary for the “origin of agency” arising from inanimate particles; but we can nonetheless quantify the extent to which a system is more agential than its parts (regardless of whether the parts, or the system as a whole, are considered to pass some absolute threshold). Specifically, if a system at a higher level of organization causes the parts it contains to do what they “normally do” *but more*, as if the complex constraints between them (that would have otherwise prevented this) were alleviated, then that system is agential (with respect to its parts).

This concept is informational insomuch as it suggests an agent has internal organization that contains information about the external environment. It is related to but distinct from Friston’s variational free-energy (Friston et al. 2007; Schwartenbeck et al. 2013), however, because it addresses information that makes its parts less frustrated (resolves conflict between them) given an environment, rather than information that minimizes surprise for the system about an environment.

Note that this notion of agency is not in any way dependent on biological fitness or selection-based notions of goals. If one were inclined to define normative behaviors for the organs of a multicellular organism, then one can assess the extent to which the organism is more agential than its organs in relation to those definitions. But if one insists that biological agency is only meaningful in the specific sense that pertains to natural selection, then that is easily accommodated. This approach may well be more natural, when applied at the level of cells (rather than organs), where an evolutionary history coincides with this level of agency as an individual. For example, suppose we assume that individual cells have the goal of surviving and reproducing, i.e., that is what they normally do. But they cannot all do that simultaneously very well because of the constraints between them, such as limited resources and other conflicts of interest. Then multicellular agents at a higher level of organization differ from disorganized cell colonies because their organization causes the cells they contain “to do better at what they normally do” despite the constraints between them. For example, an organism may do better than you would expect from the local equilibrium of a (disorganized) cell ecology (the organism is, after all, an *organized* cell ecology). The organization of the organism constitutes knowledge of the world insomuch as it enables it to forgo short-term gains and thereby attain greater long-term gains. This seems biologically meaningful since single-celled organisms do not normally grow into colonies of tens of trillions that survive for decades on dry land, for example. Moreover, the specific arrangements of cells involved in multicellular organisms involves a developmental process which controls and regulates differential reproduction of somatic cells. Note that this approach ascribes agency at the higher level of organization without assuming that surviving or reproducing is a goal *for the multicellular organism*. Its agency is defined in relation to the agency of its parts. Of course, our approach also means that agency of the organism can be different or absent if we define the agency of the parts differently.

The result is a concept of agency that relates directly to the ability of a system to problem-solve—specifically, to find (good) solutions to combinatorial constraint optimization problems. This “intelligent agent” sense of agency (Okasha 2023) may seem too special for many types of biological systems of interest. But problem-solving, construed in an appropriate space of possibilities, can be identified as a fundamental feature of all organisms (Fields and Levin 2022). An important difference from previous work is that this improvement in behavior can be quantified not against an arbitrary observer-defined objective function, but against the objective function defined by resolving the constraints among the parts.

## A Goal of One’s Own

We arrive at a notion of agency based on the idea of a system that exhibits goal-directed behavior that is greater than that of its parts. But how might a system acquire the necessary organization to exhibit agency in this sense? If, for example, the parts are placed into an organization that causes them to better resolve their mutual constraints, and this organizational structure is mandated by some sort of top-down extrinsic conditions or factors (e.g., a designer), is that still an agent? In other words, does the manner of its origination matter? Perhaps we might want to restrict a definition to systems that are *able to acquire* an internal organization that exhibits goal directed behavior that is greater than that of its parts. Notice that the identity of the agent is slipping from one level of organization to another and it might be more useful to state this in an explicitly bottom-up manner: a system of parts is agential if *they are* able to acquire organized relationships among themselves that exhibit goal-directed behavior greater than that which they exhibit as individuals (i.e., before the formation of those relationships) (Watson et al. 2022). Is it at least possible in principle that a system acquires the necessary organization to be agential *for itself*—in other words, to *create itself?* Otherwise, a notion of an agent with intelligence that must be designed-in is of little use to biology.

Concretely, we can focus on how the energy function of a dynamical system can be modified such that it improves its outcomes with respect to the original objective function. Where might such a favorably modified energy function come from? Explaining how this might be possible depends on describing a mechanism by which a landscape becomes “deformed” in a manner that confers such goal-directed behavior or improvement in problem-solving ability. We describe one such mechanism that has been previously presented (Watson et al. 2011a, c), not to suggest a general solution to the agency problem, but just to show that such a mechanism is not wholly mysterious. This is a simple bottom-up mechanism, describing how short-sighted self-interested parts change their relationships with one another (Watson et al. 2011c). The effect of this is equivalent to a distributed and unsupervised associative learning mechanism. This does not depend on a training regime or past experience at the system level that has any foreknowledge of the objective function. First we describe this as a distributed learning mechanism (i.e., helping ourselves to a distributed, unsupervised learning mechanism), then we discuss how this type of learning is natural in certain kinds of dynamical systems (e.g., in networks that are unconventional learning systems).

### Learning to be an Agent

In the example of a neural network learning XOR (Fig. 1, Box 1, Box 2), there was no constraint-solving implied, just the learning of a given input–output mapping. However, in other examples, a neural network can also define a problem-solving process (Hopfield and Tank 1985). In this case, the initial weights of the network encode the objective function (as an energy function), and running the network from a random initial condition finds a locally optimal solution to that problem. If the system is subject to occasional disturbances (e.g., shocks or perturbations that randomize the states, but not the weights), the system will visit a distribution of such locally optimal attractors. These are still only locally optimal solutions, however (so no agency implied). In general, if the constraints are low order (e.g., pairwise), there will be a positive correlation between the quality of a solution and the size of its basin of attraction (i.e., the number of initial conditions that lead to it) (Watson et al. 2011a). Now, if the system modifies the weights of the connections, this changes the energy function of its dynamics—*System2* deviates from *System1*. But how should this modification be made (without design or a benevolent teacher) such that *System2* finds better solutions than *System1*?

**Fig. 2:**
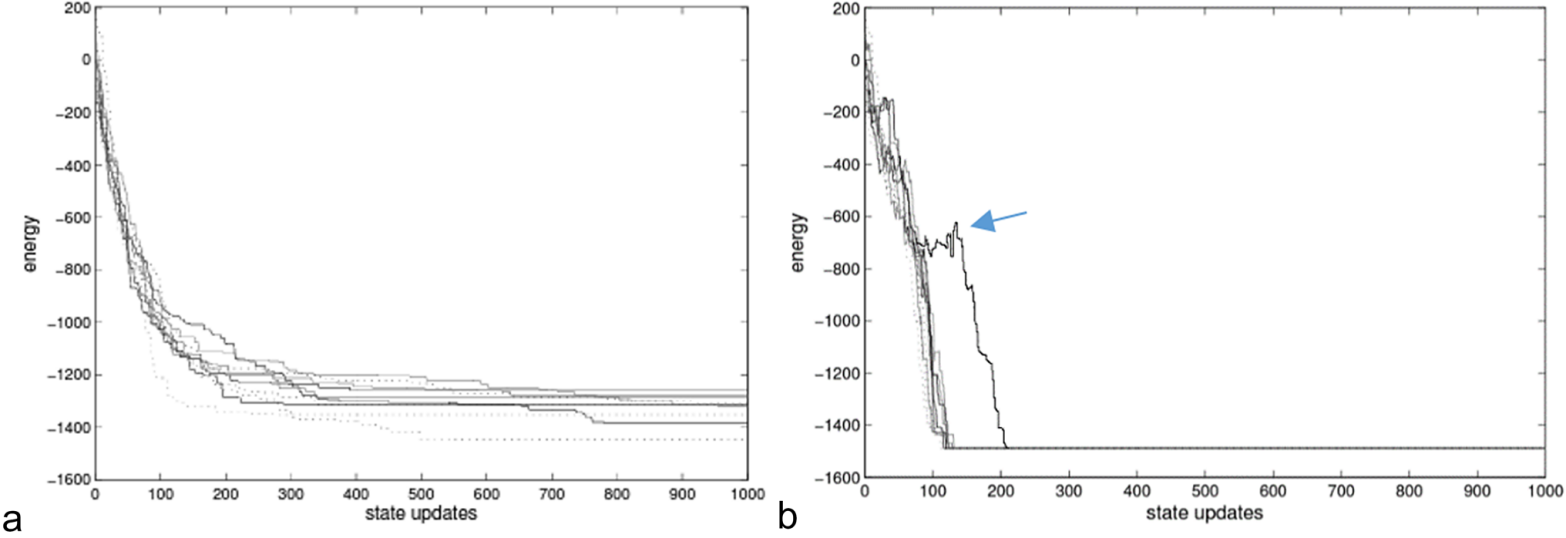
Adapted from Watson et al. (2011a). **a** The inexperienced system always goes down in energy according to local gradients (but gets stuck in local minima of variable quality). **b** The experienced system (i.e., after learning) sometimes goes up (transiently) in specific ways that enable it to go down further in the long term. One particular example trajectory shows quite a long upward excursion (arrow)

It has been shown that this modification can be provided by unsupervised Hebbian learning—which requires no external teacher or reinforcement signal—but simply applies a positive feedback on observed correlations (Watson et al. 2011a ; Buckley et al. 2023 in prep). Specifically, the system is allowed to spend most of its time at local attractors, with occasional shocks that cause it to visit a distribution of attractors over time. Over a timescale where many such attractors are visited, the weights of the network are slightly modified by Hebbian learning, and the network slowly learns an associative model of the configurations that it visits. We call this a “self-modelling” dynamical system—a dynamical system with a behavior that is augmented by a model of its own past behavior (i.e., by knowledge of its own behavior and the internal states it experiences given an environment) (Watson et al. 2011a). Under these conditions, the attractors of *System2* relate to *Function1* in a specific way. Specifically, it can be observed that the basins of attraction for attractors of *System1* that are visited most often are enlarged at the expense of attractors that are visited less often. Since larger attractor basins tend to correspond to higher-quality solutions, the behavior of *System2* deviates from *System1* in a manner that finds the high-quality solutions more often (Fig. 2).

An organization that improves the ability of a system to resolve its own constraints in this way can be *conservative* or also *innovative*. By a conservative organization we mean one that holds on to good states already identified, i.e., increasing the basin of attraction for visiting good solutions previously discovered in the past. An innovative organization, in contrast, can also promote good states that are novel—that have not previously been visited in past experience. In contrast to mere solution recall, innovation of this kind is required for genuine problem-solving or constraint optimization (likewise, for the evolution of evolvability and nonrandom evolutionary innovation; Pigliucci 2008; Kounios et al. 2016; Watson 2021). This kind of innovation is a type of *generalization* (Fields and Levin 2022), i.e., the ability to respond correctly to novel inputs or generate (nonrandom) novel patterns (Fig. 1, Box 1, Box 2).

Box 1: Non-decomposable functions and the under-determination of component organization Each unit in a Perceptron network produces a high output value if the weighted sum of its inputs is sufficiently high. Each unit thus defines a linear decision boundary in the feature space (dotted lines, Fig. 1). Since there is no single straight line that separates both of the positive samples from both of the negative samples, the XOR function is “nonlinearly-separable,” and correct classification is not possible with one unit. However, Fig. 1.ii shows an arrangement of multiple units that can classify the four training samples correctly. Here hidden unit h1 computes the decision boundary h1 = B-AND-NOT-A and h2 = A-AND-NOT-B (and these are combined with output = h1-OR-h2). Another possibility is to exchange the roles of the two hidden nodes, such that h2 = B-AND-NOT-A and h1 = A-AND-NOT-B. A third, structurally different solution is shown in Fig. 1.iii. Here h1 = A-OR-B, h2 = NOT(A-AND-B) (and output = h1-AND-h2). All of these can be reasonable explanations of *how* A XOR B is computed by a network from examination of its parts *in a particular instance of a network*. That is, if we take for granted the particular organization presented, the particular decomposition of the function as a whole into the functions of the parts, then it is easy to provide a component-to-system account of how that function is performed.

However, this example also shows that correct classification of the training samples *under-determines* the internal architecture (i.e., there are many such organizations that classify with zero error). Thus to answer the question why does h1, for example, compute B-AND-NOT-A (rather than A-AND-NOT-B, A-OR-B, NOT(A-AND-B), etc.) we need to refer to what the other parts are doing—a particular context (using one part to explain the form taken by another part). Note that an “on average over all possible contexts” qualifier is not sufficient to rescue an explanation of *h1* that is independent of context (neither the input A nor the input B has any information about the output on average). In fact, of the 8 possible functions a node can compute that have sensitivity to both inputs, either hidden node can be any of these 8 and the network can still classify the samples correctly. So, this example shows that it is possible that a system (of only two components) computes some function, and we can easily explain how it achieves zero error on the training samples (for a given system organization), but knowing this tells us absolutely nothing about what any of its components computes.

Box 2: System learning cannot always be decomposed into the learning of each part A historical explanation of acquired structure, such as an evolutionary account, finds in neural networks an analogue in the learning process (Watson and Szathmary 2016). We might say, for example, that the “ultimate cause” of this network organization is that it reduced error on the target output function. Narrowing the scope of the historical account to address the parts rather than the whole might be tempting; for example, the changes to the weighted connections entering h1, reduced the error on the output *ceteris paribus*. However, if an explanation requires reference to context, and that context is itself dependent on the thing we are trying to explain (i.e., h1 depends on h2, and h2 depends on h1), the historical causal explanation of the whole cannot be broken down into historical causal explanations of each part. Thus, over the multiple internal organizations that classify the samples correctly, the training samples do not answer the question of why the parts have the particular form they do (Box 1). Although this internal variability does not affect the training error, these different arrangements compute different functions (i.e., shaded areas are not the same). This matters to how a network generalizes—the outputs that it gives for inputs it has *not* seen during training. For example, point “x” (Fig. 1.iv) is classified the opposite way by the two networks. So, if the training error does not distinguish them, what explains the function that the network computes?

Actually, what happens as a neural network is learning to solve XOR is quite interesting. Note that in either one solution (either Fig. 1.ii or Fig. 1.iii) the decision boundaries of the two hidden units are the complement of each other (reflections about the center of feature space). During learning there is a symmetry-breaking phase where the roles of h1 and h2 mutually define one another, settling on one of the two possible mutually-exclusive system organizations. This dynamic has sensitive dependence on initial conditions. If h1 deviates slightly toward B-AND-NOT-A then h2 favors A-AND-NOT-B, and the first network arrangement obtains. Conversely, if h1 deviates slightly toward A-OR-B, then h2 favors NOT(A-AND-B), and the second network arrangement obtains. That symmetry-breaking dynamic is fundamentally systemic—involving both parts (and the output) simultaneously. It is better understood as a process that relieves internal *stress between the parts* caused by the environment than a process that is determined by the environment (error signal) per se.

Although the internal organization is therefore not determined by the training function, it is exactly this organization that determines how the system generalizes, i.e., how it induces a general rule from specific examples. Generalization is essential for a learning system to make a specific prediction about a new input it has not encountered before (by definition). But over the set of all possible models consistent with the training data, all predictions are possible (precisely because the training data did not specify what the output should be). Thus, to make a prediction (to do anything beyond memorizing the training data), a learning system *must* therefore adopt a specific internal architecture which will determine how it generalizes. Thus, whilst there remains a microscale explanation for how any particular network computes the function that it does (given a particular organization), neither the parts, its initial construction, nor the training environment explains why it computes the function that it does. Furthermore, initial differences between weights account for very little of the final differences compared to the systemic symmetry-breaking dynamics. Thus the function of a part is more strongly determined by internal complementarity with other parts than it is by its own initial conditions or the training samples. The organization of the system and what it computes thus depends intimately on *the history of the system, the whole system, and nothing but the system*. This observation is useful for clarifying what it might mean for a system’s behavior to be determined by the system itself—not by the internal parts of which it is composed nor by the external training or selection context it is in. This suggests that the important point is not just that a system’s organization can be under-determined, but that such under-determination, a fundamental characteristic of inductive learning, is fundamental for a system to have behaviors *of its own*.

The role of generalization is critical. Learning is not just about doing well in the environments already experienced (i.e., the training data) but, more importantly, learning requires generalization from past experience to new environments (i.e., the test data including previously unseen examples). The response to supervision (or reinforcement) with respect to past data cannot explain the generalization that a system exhibits, i.e., the system has no basis in the data to prefer one response to a novel input over any other response. But if a system does not represent such a preference it cannot generalize at all; at best, it can only memorize and recall the past. Doing well on past samples therefore has no explanatory power to account for how the system generalizes on new samples. Accordingly, although the internal organization of the agent is under-determined by the external input–output relation that was reinforced by past experience, the difference between one particular internal organization and another is precisely the difference between an agent that problem solves and a system that merely memorizes past solutions. Since the internal organization necessary for generalization is not determined by its past experience (by definition), the problem-solving ability of an agent, such as it may be, belongs properly to the systemic history of the agent and not to its component parts (nor its training or environment).

### Learning in Nonneural Substrates

Given that they are the substrate of conventional intelligent agents, it is perhaps not surprising that neural networks can learn to solve problems better with experience (even if it is surprising that this can arise from fully distributed and unsupervised learning mechanisms). We might imagine that this is because the learning mechanisms of neural networks are selected for the purpose of such problem solving. However, the same learning principles may obtain much more generally in biological systems.

Specifically, the same kind of information gain is possible through bottom-up processes, i.e., without presupposing a learning mechanism that is selected (or designed) for the purpose of such innovation (Watson et al. 2014, 2016; Watson and Szathmary 2016).^5^ For example, a simple model of a social network shows how a number of these ideas fit together (Davies et al. 2011). When a system of simple utility-maximizing agents acts within a network of interagent constraints, their conflicting interests generally prevent them from finding configurations that globally maximize total utility (Davies et al. 2011; Watson et al. 2011c). The equilibria they find are merely local optima in total utility. However, under some conditions, if they are able to adjust their social relationships with one another, e.g., by allowing them to pay more attention to some relationships than others (consistent with their *individual* self-interest), this changes their dynamical trajectories and the configurations to which the system is attracted. Specifically, the equilibria of *System2* (with the new relationships) are not only different from the equilibria of *System1* (with the original constraints) but they are actually better solutions to those original constraints (Davies et al. 2011; Watson et al. 2011c). If we follow a trajectory of *System2* (determined by the new energy function of the system) from a random initial condition and, as it moves along this trajectory, measure the total utility of the configurations it visits with respect to the original constraints of *System1* (the objective function), we can see that it sometimes goes *down* in total utility in the short term, and in so doing arrives at points of *higher* total utility in the long term, i.e., it is goal directed with respect to the normative (utility-maximizing) behavior of its individual parts (Davies et al. 2011). In this sense, the information contained in the network of learned relationships confers agency on the system that is greater than that of the agents it contains.

Biological networks of other kinds, including evolving gene-regulatory networks and ecological networks, can exhibit this same behavior (Watson et al. 2014; Power et al. 2015). Importantly, for the ecological network the system-level ability to find states that resolve community constraints arises without any application of selection on the system as a whole, i.e., from only individual-level selection on individuals within each species (no population of ecosystems or group-level selection is required) (Power et al. 2015).

A further exploration of such ideas is provided in Tissot et al. (2023). This shows that when the network of modified interactions affects the relative timing of subagent actions (i.e., relationships determine *when* they take actions rather than directly influencing *which* actions they take), this can effect a multi-scale behavior that picks out particular subsets of agents to “work together.” This can cause them to move against their local energetic forces (short-term gains), and enable them to respond to the relatively weak signals that lead to the global optimum (greater long-term gains) (Tissot et al. 2023). This mechanism has problem-solving abilities that are significantly superior to the other models in some cases. The internal organization that is induced has the effect of rescaling the problem-solving dynamic from a gradient process in the state space of the original variables to a state space over emergent groups of variables (Watson et al. 2011b; Tissot et al. 2023). In effect, this changes the “adjacent possible,” i.e., enabling the system to “jump” to state configurations that are better solutions without visiting the poor-quality solutions in between (Tissot et al. 2023).

This notion of learned agency, and the increased problem-solving ability it confers, is therefore intrinsically cognitive (Thompson 2010; Watson et al. 2022; Watson and Levin 2023). The resulting system acquires information from past experience, internalizes it in the form of changes to internal connections (or timing dependencies), and deploys it in a manner that exhibits delayed gratification in the short term, thus facilitating the discovery of superior solutions in the long term (Watson et al. 2022).

In each case, *given the organization of the internal relationships that obtain* it is unproblematic to explain how the system arrives at the equilibria that it does in a mechanistic reductionist sense, i.e., by examining the behavior of each part in interaction with the others as we find them now. But explaining why this particular organization obtained, and not some other, is the result of subtle symmetry-breaking dynamics that belong properly to the intimate history of the system as a whole and cannot be decomposed into the parts or the training/selection of the parts (Box 2). Moreover, it is exactly the difference between this particular internal organization and the set of all such organizations consistent with the data (past experience) that confers a problem-solving advantage (innovation and not just conservative memory). In this sense, a system-to-component account is required to explain how the system came to be agential, i.e., why the parts have the particular structural relationships that they do, and how this confers a goal-directed system-level behavior.

## Discussion

### Organismic Agency

We thus arrive at a set of interdependent concepts that constitute a notion of agency and goal-directed behavior. In particular, it characterizes the agency of a biological system at one scale in comparison to the biological agency of systems at a lower scale of description (its parts) with reference to some decomposition into parts with goals of their own.

Without this perspective, the intuitive notion that organisms play an active role in the evolutionary process, and therefore require an agential account, is susceptible to the contrary position that each gene they contain evolves because of, and only because of, the fitness differential it confers—no matter how complex its selective context may be. However, this presupposes an internal organismic structure that also needs to be explained. To the extent that the phenotype of an organism (and hence its fitness) is a non-decomposable function of its genetic constituents (Watson et al. 2022), this structure is not explained by reference to the evolutionary history of each part, but demands a historical account that depends on the system as a whole (Box 2). The internal organization of an organism can be massively under-determined by its selective history but still play an important role in subsequent evolutionary trajectories. For example, the evolution of a genotype–phenotype map, as determined by a gene-regulatory network, for example, can exhibit the ability to produce multiple fit phenotypes that have been selected in the past, and also other phenotypes that have the same structural regularities (Kounios et al. 2016; Kouvaris et al. 2017). This can then accelerate the evolution of phenotypic novelty (Kounios et al. 2016) as per the intuition of facilitated variation (Gerhart and Kirschner 2007; Parter et al. 2008).

Numerous cases of agential behavior at the level of cells, organs, developmental structures (such as limb buds), and other levels of organization have been studied empirically (for example, Baluska and Levin 2016; Levin and Dennett 2020; Levin et al. 2000; Levin 2021a, b, 2022; Fields and Levin 2022). The ability of organisms and parts of organisms to exhibit goal-directed behavior in such examples can be quite extraordinary; such as maintaining tubule diameter despite massive changes in cell size, finding new pathways to regenerate a head (in 15 minutes) despite poisoning that explodes the original cells, rearrangement of facial features during metamorphosis despite unusual starting positions, and skin cell clusters that demonstrate free-swimming locomotion and other behaviors that are not part of their evolutionary history. This is not to mention the common garden-variety ability of multicellular organisms to produce reliable target form and function, at scales that are enormous compared to the cells from which they are composed (let alone the molecules), under extraordinarily varied environmental conditions and material failures. An agential approach to experimental studies in developmental biology and regenerative medicine affords results that do not seem available in conventional bottom-up approaches (Levin 2009, 2012, 2013, 2020, 2021a, b, 2022; Fields and Levin 2018; Levin and Martyniuk 2018; Fields et al. 2020,).

Some of these dynamics may depend sensitively on the symmetry-breaking dynamic that results from resolving internal stresses specific to the organism’s internal architecture (given the environment experienced). Nonetheless, it is precisely this internal organization that determines how the phenotype of the organism is anything more than a memory of past selective conditions. The generalization that is thus enabled makes the organism more than the sum of its parts in a way that can be formally quantified (given a definition of its parts) in terms of an increase in problem-solving ability characterized by the ability to avoid short-term obstacles and attain higher-quality benefits in the long-term—a hallmark of intelligence. The solutions thus found can then be leaders in the evolutionary processes acting on the parts.

Phenomena such as niche construction, inclusive inheritance, and phenotypic plasticity—modifying the selection, inheritance, and variability of phenotypes, respectively—can then be accommodated in a more fluid notion of evolutionary individuality by virtue of the fact that they cause parts to be selected, inherited, and to vary together, rather than individually (Watson et al. 2016; Watson and Szathmary 2016). The origin of novelty can also be understood, neither as a direct consequence of past selection nor merely fortuitous happenstance, but rather a (nonrandom) generalization from past experience (Kounios et al. 2016; Watson 2021). This perhaps goes some way to providing a theoretical basis for the evolution of evolvability. Without concepts of inductive learning it is hard to explain how an evolutionary process can change its own evolutionary trajectories for the better (except by a higher-level selective process) (Kounios et al. 2016), but with inductive learning this is possible. It also offers some possibilities for a theory of evolutionary transitions in individuality (Watson et al. 2022). In particular, it can potentially explain how a selection process at one level of organization can result in functional relationships that cause entities to forgo fitness benefits in the short term, without presupposing a higher-level selective process that is looking after their long-term fitness interests (Watson et al. 2022; Tissot et al. 2023).

This position suggests that intelligent problem-solving competencies are not a special case in biological systems, but rather a fundamental feature of all organisms—it is precisely what makes an organism more than the sum of its parts, and an active agent in constructing itself (Thompson 2010; Levin 2021b, 2022; Levin et al. 2021; Lyon et al. 2021; Watson et al. 2022; Watson and Levin 2023).

In conclusion, we have presented a notion of agency based on the goal-directed behavior of a system relative to the goal-directed behavior of its parts. Such behavior can be evaluated by comparing the total frustration or energy of the system at local equilibria given some original organization of the parts to that obtained with some new organization of the parts. Further we make the case that such organization can potentially be acquired by the system (without presupposing that it is already an agent). One such possibility is a mechanism of fully decentralized and unsupervised learning, motivated bottom-up by the interests of the component parts, without the need to mandate a learning mechanism designed or selected for this process. Finally, this distributed learning process, and its ability to confer goal-directed behavior on the system, may depend on an internal organization that arises through a non-decomposable symmetry-breaking process. In this sense, the agent creates itself.

## Footnotes

1 Actually, since the movement in any one unconstrained dimension only finds a local minimum, this constraint-based notion of local minima describes the difference between the summative unconstrained movements of the parts and the movement of the system. Notice that this suggests a sense in which interactions prevent the parts from doing what they would otherwise do naturally.

2 In some cases (discussed later), another possibility is to change the “adjacent possible” such that in effect it becomes possible to “jump” or “tunnel through configuration space” to a specific point in a specific direction—hence arriving at the new location without visiting the intermediate points.

3 Interestingly, in this example we can infer the values of these “internal” variables by making a few observations over time of the “external” variables—this is why we don’t usually think of momentum as an internal variable (i.e., we can observe momentum *almost* directly—we just need two observations of the external position at different times). Nonetheless, we can think of momentum as an unobservable at a given point in time. The fact that it can be inferred from external observations over time links quite neatly to the idea that a useful internal state can be induced by experience (over learning timescales).

4 As mentioned, we are working toward a *relative* notion of agency.

5 The reason that networks are important (rather than selection acting on a vector of genetic loci, or a vector of phenotypic traits each controlled by an independent genetic basis) is that networks (like neural networks) can be generalized models of past experience, not just a rote memory. Although natural selection explains why a gene-regulation network, for example, gets better at producing fit phenotypes (in the same way that supervision explains why a neural network achieves the target function), the inductive properties of the network representation can explain how its evolvability increases and improves over evolutionary time (Kounios et al. 2016), i.e., to improve its problem-solving ability—not merely to find a solution, but to get better at finding solutions.
